# *κ*/*ι*-Carrageenan Blends in Plant Capsules: Achieving Harmony Between Mechanical and Disintegration Properties

**DOI:** 10.3390/md23070284

**Published:** 2025-07-09

**Authors:** Zhenyu Liu, Chuqi He, Zhibin Yang, Qing Zhao, Yuting Dong, Jing Ye, Bingde Zheng, Ranjith Kumar Kankala, Xueqin Zhang, Meitian Xiao

**Affiliations:** 1 College of Chemical Engineering, Huaqiao University, Xiamen 361021, China; 2226202020@stu.hqu.edu.cn (Z.L.); 21011087003@stu.hqu.edu.cn (C.H.); 2326211033@stu.hqu.edu.cn (Z.Y.); 2226202044@stu.hqu.edu.cn (Q.Z.); 23011087007@stu.hqu.edu.cn (Y.D.); yejenny@hqu.edu.cn (J.Y.); bingd.zheng@hqu.edu.cn (B.Z.); ranjithkankala@hqu.edu.cn (R.K.K.); 2 Xiamen Engineering and Technological Research Center for Comprehensive Utilization of Marine Biological Resources, Xiamen 361021, China

**Keywords:** carrageenan, gel, fast-disintegration capsule

## Abstract

The fast-disintegrating capsules rapidly disintegrate in various physiological environments, ensuring therapeutic efficacy. The formulation of plant-based capsules with balanced mechanical and fast disintegration characteristics continues to present technical challenges in pharmaceutical development. In this study, natural marine polysaccharides were utilized to achieve both rapid disintegration and excellent mechanical properties by combining *κ*-Carrageenan (*κ*-C) and *ι*-Carrageenan (*ι*-C). Additionally, the selection of KCl + NaCl mixed coagulants, along with the evaluation of their types, mass fractions, and ratios, enhanced the mechanical properties and transmittance of the capsules. FTIR analysis revealed that the membrane with a 5:5 *κ*-C/*ι*-C ratio formed hydrogen bonds, which were beneficial to its fast disintegration. SEM analysis revealed a dense microstructure in this formulation, contributing to its improved mechanical properties. Finally, this study hypothesizes that the disintegration behaviors of the capsules exhibited significant pH dependence, with ion exudation predominating in pH 1.2 and pH 7.0 media, while swelling dominated under pH 4.5 and pH 6.8 media. The prepared carrageenan blend-based capsules exhibited fast disintegration properties while maintaining excellent mechanical and barrier properties, thereby broadening the application of plant-based capsules in the field of medicine.

## 1. Introduction

Hard capsules are widely used as delivery systems in the pharmaceutical and nutraceutical industries, with gelatin traditionally serving as the primary material for their construction. However, animal-derived gelatin has inherent limitations, such as restricted applicability, storage instability, and incompatibility with certain drug components [1,2]. As consumer demand for natural and health-conscious products continues to rise, plant-based hard capsules have emerged as ideal alternatives to gelatin capsules. These capsules offer the advantages of being vegan-friendly, exhibiting enhanced stability, and being strongly compatible with a wide range of formulations [3]. Currently, plant-based capsules are mainly categorized into types like HPMC, pullulan, and starch-based varieties [4]. Among these, HPMC has gained widespread attention, largely due to its stability, compatibility, and safety [5,6]. However, plant-based capsules face two critical limitations, higher prices compared to gelatin capsules and longer disintegration time, which limit their expansion in applications [7,8].

The fast-disintegrating capsules are specialized pharmaceutical formulations designed to disintegrate quickly in the stomach or intestines within 15 min, thereby facilitating the rapid release of the drugs. These capsules offer notable advantages in enhancing bioavailability, accelerating the onset of therapeutic effects, and improving patient compliance [9]. Currently, many drugs for cardiovascular emergencies and antiepileptic medications can only achieve their maximum therapeutic effects through rapid drug release. According to the United States Pharmacopeia and Chinese Pharmacopoeia (2020 edition), the disintegration times of these capsules are within 15 min in at least three different media, including hydrochloric acid (HCl) solution (pH = 1.2), buffer solution (pH = 4.5), phosphate buffer (pH = 6.8), or deionized water (pH = 7.0) [10]. The pH = 1.2 solution simulates the highly acidic gastric environment during fasting, while the pH = 4.5 solution corresponds to the postprandial gastric environment. Additionally, the pH = 6.8 and pH = 7.0 solutions simulate the intestinal and colonic environments, respectively. In certain specialized cases, such as when drugs are administered via specific routes or in patients with gastrointestinal disorders, they may be directly targeted to these positions to exert their therapeutic effects through fast-disintegrating capsules [11]. Gelatin capsules, owing to their excellent water solubility, can rapidly disintegrate in different media [12,13]. Song et al. prepared phenylboronic acid-functionalized hyaluronic acid as a gelling agent via chemical modification, successfully constructing a pH-responsive pullulan–hyaluronic acid system that enables rapid disintegration in four pH solutions [14]. Zhou et al. accomplished rapid disintegration capsules through heterogeneous hydroxypropylation for conventional carrageenan employing an hydroxypropyl carrageenan–hydroxy propyl methyl cellulose (HPMC) system [15]. To date, there have been no reports of plant-based natural polysaccharide hard capsules that can achieve fast disintegration and drug release across the solutions mentioned above.

The plant-based capsules primarily consist of three key components: film-forming agents, gelling agents, and coagulants [16]. Interestingly, gelling agents reduce solution fluidity and facilitate the formation of hydrogen bond networks, which can directly influence the mechanical properties and disintegration behavior of the capsules [17]. Coagulants (e.g., Na^+^, K^+^, and Ca^2+^) interact with gelling agents, promote the formation of hydrogen bonds through ionic cross-linking, induce conformational transitions to helical structures, and facilitate the formation of the gel network [18]. Among common gelling agents, carrageenan is extensively employed in gastro-soluble capsules due to the deprotonation of its sulfate groups and subsequent electrostatic repulsion in the gastric acid environment, which disrupts intermolecular hydrogen bonding networks and leads to rapid disintegration [19].

**Figure 1:**
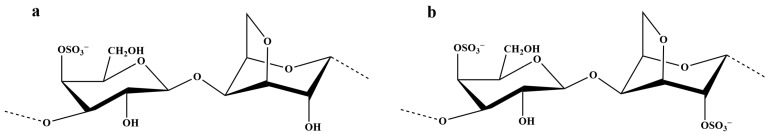
Structures of natural polysaccharides: (**a**) *κ*-Carrageenan and (**b**) *ι*-Carrageenan.

Carrageenan is a natural, water-soluble sulfated polysaccharide extracted from Rhodophyta, which are commonly found in regions of Europe and North America along the Atlantic Ocean [20]. Due to its biocompatibility, gelling capacity, bioactive properties, and general safety with no reported significant interactions with most drugs, carrageenan has emerged as a primary biomaterial for enhancing the controlled release of drugs [21,22,23,24]. It is composed of alternating *β*-D-galactose and 3,6-anhydro-D-galactose units, linked by *α*-1,3- and *β*-1,4-glycosidic bonds [25]. The position and content of sulfate groups determine the classification of carrageenan into *κ*-Carrageenan (*κ*-C), *ι*-Carrageenan (*ι*-C), among others [26]. As shown in Figure 1, *κ*-C contains one sulfate group (-OSO_3_^−^) at the C-4 position of *β*-D-galactose, while *ι*-C has two sulfate groups, one at the C-4 position of *β*-D-galactose and the other at the C-2 position of *α*-3,6-anhydro-D-galactose. The molecular chains of *κ*-C have a low content of sulfate groups, resulting in low electrostatic repulsion between the chains. This allows *κ*-C to form a tightly packed double-helical network structure under K^+^ induction via ionic bridging, which imparts excellent mechanical strength [27]. However, due to the overly compact packing of molecular chains in this network structure, the degree of freedom for segmental movement is reduced, leading to poor elasticity [28,29]. In contrast, *ι*-C contains a higher content of sulfate groups, which have extremely low pK_a_ values and remain fully ionized over a broad pH range. This extensive ionization endows *ι*-C with strong hydrophilicity [30], enabling it to rapidly absorb water and swell in acidic to neutral solutions, thereby facilitating capsule disintegration. Due to the significant repulsion between sulfate groups, the gel network formed by *ι*-C has relatively poor mechanical properties [31], limiting its practical applications in the pharmaceutical field. *κ*-C has been widely adopted as the primary gelling agent in plant-based hard capsules but achieving an optimal balance between mechanical and disintegration properties remains challenging. He et al. reported that blending *κ*-C with locust bean gum (LBG) at a ratio of 3:1 (*κ*-C/LBG) significantly enhanced the mechanical properties of the capsules while ensuring a disintegration time of less than 30 min in simulated gastric fluid [32]. Adam and colleagues developed a composite material for hard capsules by blending *κ*-C with gum Arabic. The mechanical properties were significantly improved, achieving disintegration time within 7.5 min in deionized water [33]. Similarly, Pudjiastuti and coworkers prepared *κ*-C-alginate and *κ*-C-starch hard capsules, with average disintegration times in deionized water of 12.80 min and 25.79 min, respectively [34]. Evidently, leveraging synergistic interactions among polysaccharide-based gelling agents serves as an effective strategy to enhance both the mechanical properties and disintegration performance of capsules. While these composite gelling agents can ensure satisfactory disintegration time of the capsules in a single solution, their ability to achieve fast disintegration in the above-mentioned four typical pH value solution environments has not yet been reported in the literature.

Motivated by these considerations, in this work, we hypothesized that the sulfate group content in *κ*-C and *ι*-C molecular structures, combined with the participation of coagulants (K^+^, Na^+^, and Ca^2+^), may synergistically regulate the mechanical and disintegration properties of the capsules. To validate this hypothesis, the present study systematically investigated the effects of the type and concentration of coagulants and the blending ratio of *κ*-C/*ι*-C on capsule performance through multiple analytical approaches, including Fourier-transform infrared spectroscopic (FTIR) analysis for molecular interactions, scanning electron microscopy (SEM) for microstructural observation, mechanical property testing, and in vitro disintegration evaluation under physiologically relevant conditions. The integrated results provided fundamental insights into the structure–property relationship of plant-derived polysaccharides, offering theoretical support for their scalable pharmaceutical application in plant-based fast-disintegrating hard capsule formulations.

## 2. Results and Discussion

### 2.1. Effect of Coagulant on Capsule Properties

**Figure 2:**
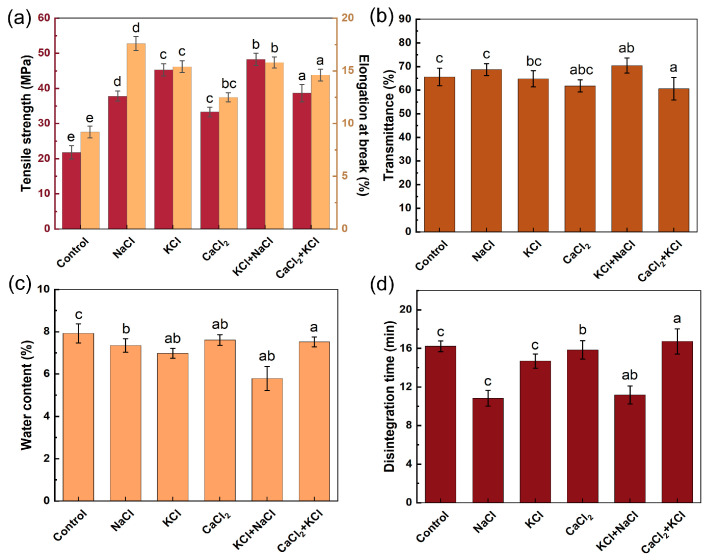
Effect of coagulant on (**a**) mechanical properties, (**b**) transmittance of membranes, (**c**) water content, and (**d**) disintegration time of capsules. Different letters above the bars indicate significant differences among groups at *p* < 0.05.

The membrane and capsules, based on the methods in Section 2.4 and Section 2.3, respectively, were used to conduct measurements for precisely evaluating the properties of HPMC-based hard capsules. The coagulant is the critical excipient of the capsules, establishing salt bridges between the colloidal particles and then prompting gel transformation [18]. Figure 2 presents the effects of varying types of coagulants on mechanical and disintegration properties evaluated at a fixed coagulant concentration of 0.14% (*w*/*w*). Figure 2a demonstrates that the membranes containing coagulants exhibited significantly enhanced mechanical properties compared to the control group (without coagulants). The membranes formulated with KCl + NaCl showed the highest tensile strength (48.26 ± 1.73 MPa), while those formulated with NaCl had the highest elongation at break (15.80 ± 0.63%). The membranes formulated with CaCl_2_ had lower tensile strength and elongation at break (33.31 ± 1.32 MPa and 12.52 ± 0.48%, respectively) compared to the other membranes containing coagulants. It was observed that the gel strength of the mixed carrageenan system induced by K^+^ was greater than that induced by Ca^2+^, which is consistent with the findings of Viet T.N.T. Bui et al. [35]. Meanwhile, it was noted that the gel formed by the interaction of Na^+^ with carrageenan exhibited good toughness. It is speculated that the combination of the enhancing effect of K^+^ on gel strength and the improving effect of Na^+^ on gel toughness may bring the mixed system to a balanced state in terms of mechanical properties [27,36]. As depicted in Figure 2b, the transmittance of all membranes surpassed 60%, among which that formulated with KCl + NaCl achieved optimal transmission performance (70.67 ± 2.71%). The water content of the prepared capsules was below 8% (Figure 2c), meeting the requirements of the Chinese Pharmacopoeia. The capsules formulated with KCl and NaCl exhibited the lowest water content (5.71 ± 0.57%), with stable storage properties. As shown in Figure 2d, the capsules prepared with NaCl, KCl, and KCl + NaCl exhibited disintegration times of 10.83 ± 0.25 min, 14.69 ± 0.31 min, and 11.17 ± 0.28 min, respectively, satisfying the specification for fast disintegration (≤15 min). The marked reduction in disintegration time observed with NaCl addition may be attributable to the preferential interaction between Na^+^ ions and the more hydrophilic *ι*-C, promoting the formation of a gel network dominated by *ι*-C helix [37]. Under K^+^ induction, *κ*-C molecules form a tightly packed double-helical network through ionic bridging. Although the disintegration time of capsules prepared with KCl is slightly longer than that of those prepared with NaCl, it still satisfies the fast disintegration specification [31,38]. These results indicated that the capsules incorporating the mixed coagulant of KCl and NaCl exhibited optimal performance in terms of mechanical, storage, and disintegration properties.

**Figure 3:**
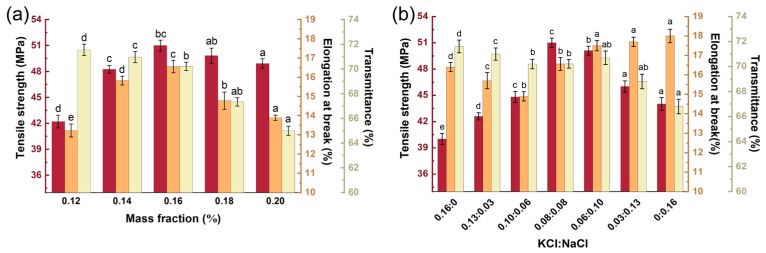
Effects of (**a**) mass fraction of coagulant and (**b**) mixed coagulant ratio on mechanical properties of membrane. Different letters above the bars indicate significant differences among groups at *p* < 0.05.

Based on the findings mentioned above, KCl and NaCl were selected as the coagulants for further concentration optimization studies. The tensile strength and elongation at break of the membranes initially increased and subsequently decreased with an increasing mass fraction of the mixed coagulant (KCl + NaCl), achieving maximum values of 51.00 ± 0.83 MPa and 16.56 ± 0.35%, respectively, at a mass fraction of 0.16% (Figure 3a). The increasing trend of mechanical properties may be attributed to the cationic promotion of gel network formation, thereby enhancing structural stability [39]. The decline in mechanical properties at concentrations exceeding 0.16% could result from the excessive addition of cations, which shield the sulfate groups on helices, thereby attenuating interactions between macromolecular chains and ultimately decreasing the value [40]. The transmittance of membranes showed a decreasing trend with an increase in the coagulant mass fraction (Figure 3a). The enhanced crystallinity induced by increasing the cation mass fraction could result in a reduction in compatibility within the system, thereby reducing transmittance [41]. The mixed coagulant (KCl + NaCl) at a concentration of 0.16% (*w*/*w*) exhibited optimal mechanical properties while maintaining a relatively high transmittance.

Based on these results, the mechanical properties were investigated by varying the ratio of the mixed coagulant (KCl + NaCl) (Figure 3b). As the ratio of KCl to NaCl decreased, the tensile strength initially increased and then decreased, while the elongation at break showed an inverse trend. The membranes demonstrated optimal tensile strength at a KCl:NaCl ratio ranging from 0.08:0.08 to 0.06:0.10 (*w*/*w*), while exhibiting maximal elongation at break within the 0.06:0.10 to 0.00:0.16 (*w*/*w*) ratio range. The transmittance displayed an inverse correlation with NaCl content, decreasing progressively. In conclusion, the membrane at a ratio of 0.06:0.10 (KCl:NaCl) exhibited the best comprehensive performance. These results supported the implementation of a KCl + NaCl binary coagulant system with an optimal total content of 0.16% (*w*/*w*) at a KCl:NaCl mass ratio of 0.06:0.10.

### 2.2. Rheological Properties of Gel Solution

The sulfate group of carrageenan affects its gelling properties, with a higher content of sulfate groups resulting in poorer gelling properties of its gel structure [42]. Thus, gels formed by *κ*-C exhibit higher gel strength but lower elasticity, whereas gels formed by *ι*-C demonstrate lower gel strength yet greater softness and elasticity. Capsules formed by a single gelling agent often fail to meet requirements. Therefore, blending *κ*-C with *ι*-C emerges as a viable strategy for optimizing the composite gelling agent. The following study investigates the impact of the *κ*-C/*ι*-C ratio on the performance of capsules through rheological analysis, tensile testing, barrier property testing, SEM, FTIR, and thermogravimetric analysis (TGA).

**Figure 4:**
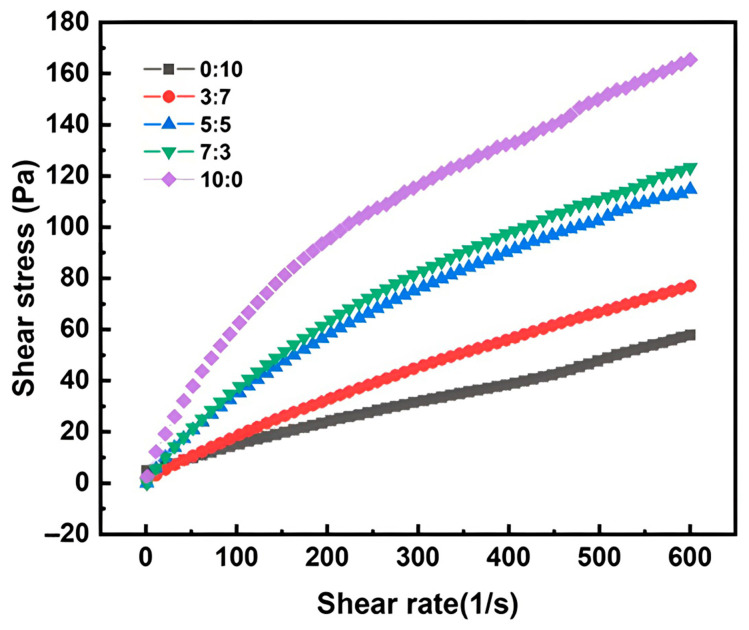
Shear stress–shear rate curves of gel solution with different ratios of *κ*-C/*ι*-C.

Firstly, the rheological properties of the gel solution are vital in evaluating whether the capsules can be molded smoothly during the dipping process. The gel solution with a varying ratio of *κ*-C/*ι*-C, prepared as described in Section 2.2, was formulated to investigate the effect of the *κ*-C/*ι*-C ratio on the rheological properties. The shear stress–shear rate curves of gel solutions are presented in Figure 4. With the increase in κ-C content, the shear stress at the same shear rate gradually increased. The increase in shear stress can be attributed to the denser chain entanglement and the formation of more binding sites by the higher κ-C content, which requires a greater shear force to disrupt, thereby enhancing shear resistance. When the ratio of *κ*-C/*ι*-C was 0:10 and 3:7, their curves exhibited near-linear relationships with relatively small slopes, indicating Newtonian fluid flow behavior with low viscosity, which led to excessively high fluidity [43]. During the dipping process, this high fluidity may impede rapid gelation and formation within the mold, resulting in non-uniform gel distribution that compromises both shape retention and thickness consistency, thereby adversely affecting the quality of capsule production. When the ratio of *κ*-C/*ι*-C was 10:0, the shear stress reached the maximum value. The curves showed non-smooth characteristics and unstable shear stress, indicating that the gel solution had excessively high viscosity [44], which resulted in poor flowability of the gel during the dipping process and thus led to excessively thick capsules. In contrast, when the *κ*-C/*ι*-C ratio was in the range of 5:5 and 7:3, the rheological properties of the gel solution showed higher stability, enabling optimal utilization of the synergistic gelling properties of *κ*-C and *ι*-C, thereby significantly improving the manufacture of capsules.

Table 1 presents the Power-law model fitting results for the data from Figure 4. All R^2^ values exceeded 0.99, demonstrating excellent conformity with the Power-law rheological model. A higher consistency coefficient (K) indicates higher viscosity and poorer fluidity of the gel solution. The n-value (flow behavior index) of the blended glue is less than 1, indicating that the gel exhibits pseudoplastic fluid behavior [45]. In general, the smaller the n, the greater the degree of pseudoplasticity of the solution and the more prone it is to shear dilution [46]. Table 1 shows that with an increase in *κ*-C content, the consistency coefficient increases while the flow index decreases. The consistency coefficient was maximum when the *κ*-C/*ι*-C ratio was 10:0, reflecting that the solution was challenging to mix uniformly and impeded cross-linking between components. Additionally, the high pseudoplastic phenomenon may cause an excessive flow of the gel solution [47], which could adversely affect the capsule preparation. When the *κ*-C/*ι*-C ratio was 0:10 and 3:7, the low consistency coefficient and pseudoplasticity could lead to phenomena such as gel slippage or failure to form a gel during the dipping process, which also could hinder capsule preparation. These results were consistent with the trends analyzed in Figure 4.

### 2.3. Mechanical and Transmittance Properties of κ-C/ι-C Membranes at Different Ratios

**Figure 5:**
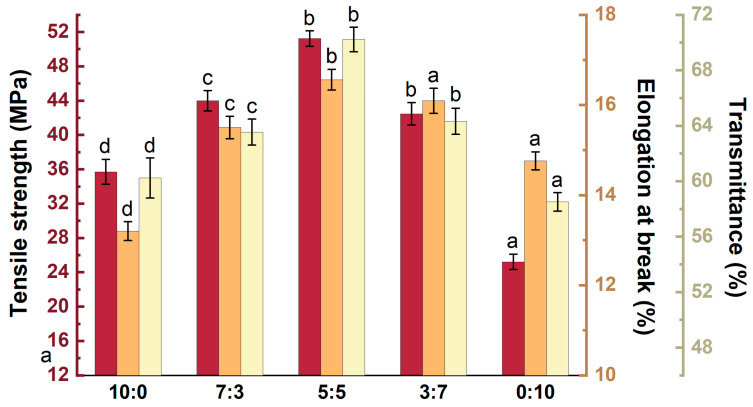
Effect of *κ*-C/*ι*-C ratio on mechanical properties and transmittance of membranes. Different letters above the bars indicate significant differences among groups at *p* < 0.05.

To further investigate the physicochemical properties of the capsule, the effect of the *κ*-C/*ι*-C ratio on the mechanical and transmittance properties of the membrane was initially investigated. As shown in Figure 5, the membrane of *κ*-C exhibited the lowest elongation at break (13.20 ± 1.12%), and the membrane of *ι*-C displayed the lowest tensile strength (25.21 ± 1.67 MPa). With increasing *ι*-C content, both the mechanical properties and transmittance of the membranes first increased and then decreased. At a *κ*-C/*ι*-C ratio of 5:5, the mixed membrane achieved optimal overall performance, with tensile strength and elongation at break reaching 51.23 ± 2.12 MPa and 17.50 ± 0.34%, respectively, and a transmittance of 70.70 ± 1.64%. At the *κ*-C/*ι*-C ratio less than 5:5, the number of sulfate groups increased, leading to enhanced repulsive forces between polymer chains. These results could be attributed to *ι*-C possessing two sulfate groups, generating stronger electrostatic repulsion between double helices compared to *κ*-C [19]. The enhanced repulsion could make the blend system looser, thereby reducing its structural stability and compatibility. Consequently, both the mechanical and transmittance properties of the membranes at these ratios were significantly compromised.

### 2.4. Barrier Properties and Hygroscopicity Properties of κ-C/ι-C Membranes

**Figure 6:**
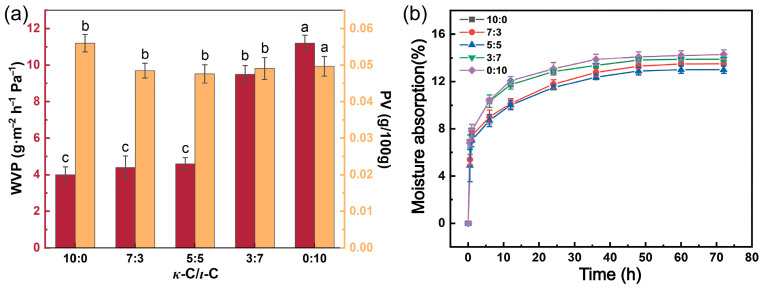
(**a**) Barrier properties and (**b**) hygroscopicity rates at 75% relative humidity of membranes at various *κ*-C/*ι*-C ratios. Different letters above the bars indicate significant differences among groups at *p* < 0.05.

The water vapor permeability (WVP), oxygen barrier property (PV), and hygroscopicity of membranes directly influence the quality of the encapsulated drugs. The reduction in these values can inhibit the erosion of the drug by environmental moisture and oxidation, thus improving the stability of the drug and prolonging its shelf life [48]. Figure 6a demonstrates the WVP and PV data for different ratios of *κ*-C/*ι*-C membranes. At the *κ*-C/*ι*-C ratios in the range of 0:10 to 5:5, the membrane exhibited a low WVP value. Notably, membranes with *κ*-C/*ι*-C ratios of 3:7 and 0:10 showed higher WVP values. These results could be attributed to the excessive *ι*-C content, where the surplus sulfate groups could enhance hydrophilicity and lead to a less dense membrane structure [49]. The PV value of mixed membranes is lower than 0.060, indicating that these capsules had qualified oxygen barrier properties [50]. As shown in Figure 6a, the PV of the membranes exhibited a trend of decreasing followed by increasing with increasing *ι*-C content, reaching a minimum value of 0.0476 g/100 g at a *κ*-C/*ι*-C ratio of 5:5, thus demonstrating optimal oxygen barrier performance. Figure 6b presents the hygroscopicity curves of *κ*-C/*ι*-C membranes with different ratios at a relative humidity of 75%. All mixed membranes exhibited consistent trends in hygroscopicity. Initially, the hygroscopicity rate increased rapidly over 6 h due to the significant humidity gradient between the dry membranes and the environment. As time prolonged, the moisture absorption of the membrane gradually plateaued due to the diminished moisture content gradient between the membrane and the ambient environment. The value of moisture absorption was stabilized after 36 h. Notably, the 5:5 *κ*-C/*ι*-C ratio membrane exhibited the lowest hygroscopicity, with a moisture absorption of 13.01%. This is attributed to the synergistic network formed by *κ*-C and *ι*-C at equimolar ratios, which enhances cross-linking density and reduces hydrophilic site exposure [35,51]. Together, the 5:5 *κ*-C/*ι*-C ratio membrane exhibited favorable barrier and hygroscopicity properties, making it more suitable for capsule storage and drug stabilization.

### 2.5. Disintegration Time

**Figure 7:**
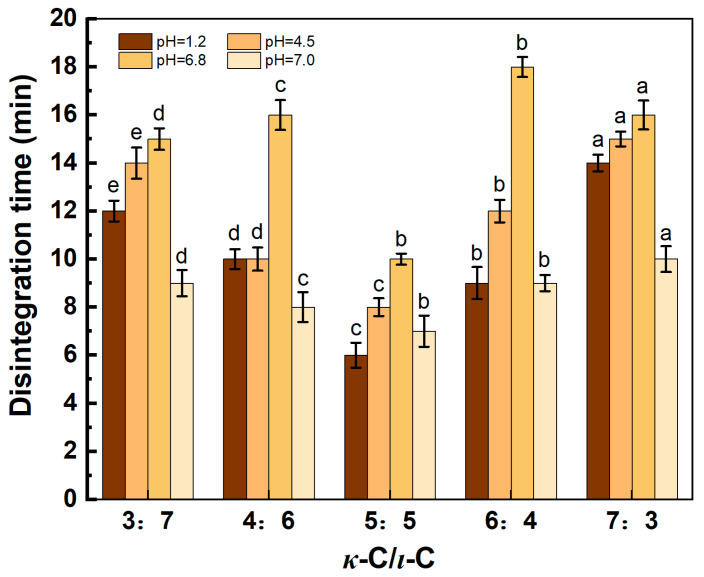
Disintegration time of capsules with different *κ*-C/*ι*-C ratios in media of pH = 1.2, 4.5, 6.8, and 7.0. Different letters above the bars indicate significant differences among groups at *p* < 0.05.

To investigate the disintegration characteristics of capsules under simulated physiological conditions, the in vitro disintegration times of *κ*-C/*ι*-C capsules with varying ratios in four different pH media (1.2, 4.5, 6.8, and 7.0) are presented in Figure 7. As depicted in Figure 7, the capsules exhibited the maximum disintegration time in pH 6.8 media, followed by pH 4.5, then pH 1.2, with the minimal disintegration time recorded in pH 7.0 media. As shown in Figure 7, the disintegration times of these capsules in pH 6.8 and 4.5 are generally longer compared to other media, which may be attributed to the K+ and Na+ ions in these media forming a concentration gradient. These metal ions may interact with *κ*-C and *ι*-C molecules, preventing the depolymerization of the helical structure and thereby delaying the disintegration time of the capsules. At a *κ*-C/*ι*-C ratio of 5:5, the capsules exhibited optimal disintegration performance, with disintegration times in all four media being less than 15 min, meeting the criteria for fast disintegration. These attributes addressed the gaps in previous studies and achieved fast disintegration in all four media [32,33,34]. The capsules at this ratio achieved an optimal balance between mechanical and disintegration properties, which were subsequently investigated through TGA, FTIR, and SEM analyses.

### 2.6. Thermal Stability of κ-C/ι-C Membranes

**Figure 8:**
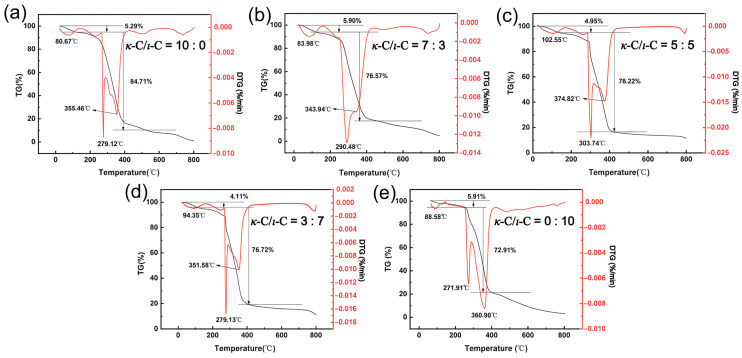
TGA curves of mixed membranes with different *κ*-C/*ι*-C ratios: (**a**) 10:0, (**b**) 7:3, (**c**) 5:5, (**d**) 3:7, (**e**) 0:10.

To investigate the effect of different *κ*-C/*ι*-C ratios on the thermal stability of membranes, TGA was conducted. As shown in Figure 8, the TGA curves of the membranes are primarily divided into three distinct stages. The first stage occurred at approximately 100 °C, where a mass loss was attributed to the evaporation of free water and the detachment of bound water due to hydrogen bond cleavage, referred to as the dehydration temperature. The second stage, occurring between 270 and 380 °C, involved significant mass loss resulting from the cleavage and rearrangement of chemical bonds in carrageenan and HPMC molecules, followed by degradation into volatile small molecules, referred to as the thermal decomposition temperature [52]. The third stage, spanning 500–800 °C, was characterized by continuous mass reduction as residual carbon skeletons from prior degradation underwent carbonization and oxidation, releasing CO_2_ [53]. In the TGA curves, both dehydration and thermal decomposition temperatures exhibited a trend of increasing followed by decreasing with increasing *ι*-C content. They reached their highest point at a *κ*-C/*ι*-C ratio of 5:5, with temperatures of 102.55 °C and 303.74 °C, respectively. The highest dehydration temperature may be attributed to the stronger hydrogen bond interaction in the gel network at this ratio, resulting in reduced water mobility and enhanced water–molecular interactions, thereby improving thermal stability [54]. The highest thermal decomposition temperature may be attributed to stronger interactions between polysaccharide chains, suggesting a better synergistic effect. Therefore, the membrane with a 5:5 *κ*-C/*ι*-C ratio exhibited better thermal stability, thereby enhancing the storage stability of the capsules.

### 2.7. Microstructure of κ-C/ι-C Freeze-Dried Membranes

**Figure 9:**
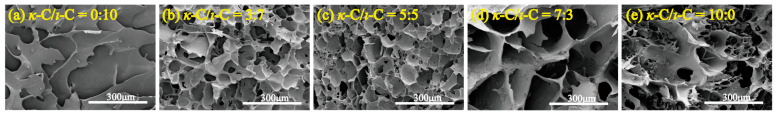
SEM images of membrane sections with various *κ*-C/*ι*-C ratios.

To investigate the effect of the *κ*-C/*ι*-C membrane structure on mechanical properties and disintegration performance, SEM was used to examine the microstructure of *κ*-C/*ι*-C mixed membranes with varying ratios. As shown in Figure 9, all cross-sectional images displayed a porous network structure. Notably, the freeze-dried membrane with a 5:5 *κ*-C/*ι*-C ratio exhibited the most compact and uniform network, characterized by smaller pore size and more consistent distribution (details in Figure S1). From a mechanical standpoint, such a compact and uniform structure facilitates the homogeneous transmission and distribution of external forces across the membrane matrix. The homogeneous pore architecture effectively redistributes mechanical stresses under applied loading, thereby enhancing the mechanical strength and deformation resistance of the capsule [55]. From a disintegration behavior perspective, the uniform porous structure may facilitate both enhanced water molecule penetration and the even release of ions during the disintegration process, thereby accelerating capsule disintegration. In contrast, membranes with relatively loose structures may contain locally dense regions that hinder ion diffusion, resulting in a prolonged overall disintegration time (Figure 7) [56]. Therefore, the 5:5 *κ*-C/*ι*-C ratio membrane exhibits a network structure that may facilitate fast disintegration while enhancing mechanical properties.

### 2.8. FTIR Spectroscopy Analysis

**Figure 10:**
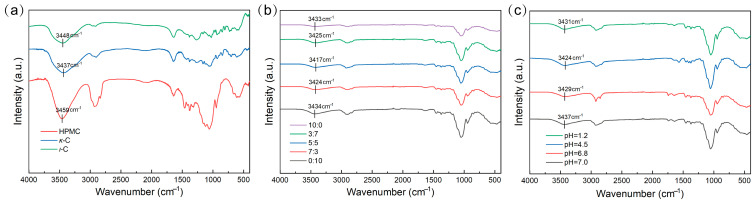
FTIR spectra of (**a**) *ι*-C, *κ*-C, HPMC, (**b**) mixed membranes with varying *κ*/*ι*-C ratios, and (**c**) freeze-dried capsules with 5:5 (*κ*-C/*ι*-C) formulation at four pH values near disintegration.

When exposed to water media, water molecules preferentially infiltrate the membrane matrix and bind to polar functional groups within the hydrogen bond network, triggering rapid swelling [57]. The disintegration times of the capsules are supposed to be related to the hydrogen bond density within the gel network. During this process, water intercalation could disrupt the initial hydrogen bonds of the capsules, weakening intermolecular forces, causing structural relaxation, and accelerating the disintegration process. To investigate the hydrogen bond content in gel networks with different *κ*-C/*ι*-C ratios, ATR-FTIR spectroscopy was utilized on *κ*-C, *ι*-C, HPMC, and mixed membranes with varying *κ*-C/*ι*-C ratios (Figure 10). As shown in Figure 10a, the FTIR spectrum of *ι*-C, *κ*-C, and HPMC showed peaks at 3448 cm^−1^, 3437 cm^−1^, and 3459 cm^−1^, respectively, which could be attributed to the stretching vibration of O-H [58,59]. The FTIR spectra of mixed membranes showed that the position of O-H shifted to lower wavenumbers at varying ratios of *κ*-C/*ι*-C, suggesting enhanced hydrogen bonding interactions involving both O-H···OSO_3_^−^ and O-H···O-H associations (Figure 10b) [58,60]. The sulfate ester groups appear in the 1250–1050 range, and the sugar rings appear in the 1100–1000 range; however, these peaks show no significant changes. FTIR analysis revealed that the hydroxyl stretching band for the 5:5 (*κ*-C/*ι*-C) formulation was significantly red-shifted to 3417 cm^−1^. These findings indicated that the intermolecular hydrogen bond interaction was enhanced, which might be the reason for its excellent thermal stability relative to other ratios [61]. These formed hydrogen bonds may also explain the rapid disintegration performance of the capsules. Figure 10c shows the FTIR spectra of the freeze-dried capsules with a 5:5 (*κ*-C/*ι*-C) formulation at four pH values near disintegration. It was observed that the hydroxyl stretching band in all solutions blue-shifted, possibly due to the breakage of hydrogen bonds during the disintegration process [62], indicating that hydrogen bonds played a crucial role in the rapid disintegration.

### 2.9. Morphological Behavior During Disintegration

**Figure 11:**
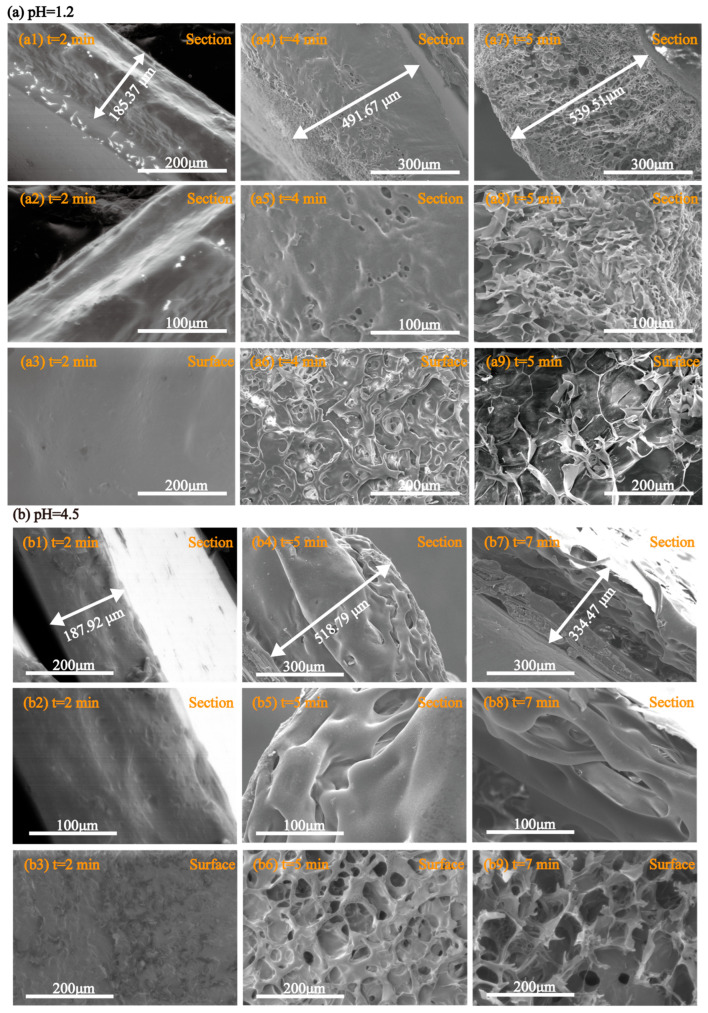

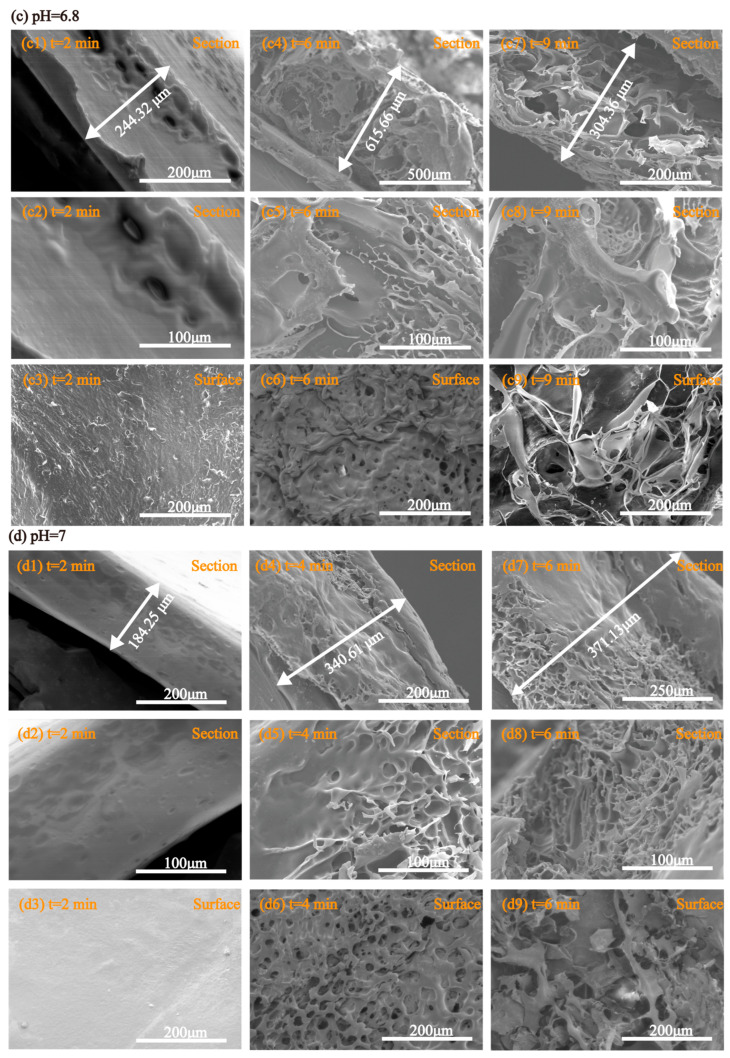
SEM images of disintegration process of *κ*-C/*ι*-C ratio of 5:5 membranes in four different media: (**a**) pH = 1.2, (**b**) pH = 4.5, (**c**) pH = 6.8, (**d**) pH = 7.0.

To investigate the disintegration behavior of capsules during the disintegration process, the morphology of capsules at a 5:5 (*κ*-C/*ι*-C) ratio was measured at initial, intermediate, and near-dissolution periods in pH media (pH = 1.2, 4.5, 6.8, and 7.0). SEM images were observed under the optimal magnification scale to ensure clear visualization of microstructural details. Figure 11 presents the SEM images of the cross-section and surface morphology of the capsules during disintegration in pH media. The original thickness of the capsules before the disintegration process was 96.62 μm (details in Figure S2). At the initial disintegration stage (Figure 11(a1–a3),(b1–b3),(c1–c3),(d1–d3)), significant capsule swelling was observed across all media. The most substantial thickness increase (244.32 μm) occurred in pH = 6.8 media, confirming that swelling represents the dominant mechanism during early-stage disintegration. The SEM surface analysis revealed a distinct morphological transition in the capsules, evolving from an initially smooth topography to one exhibiting pronounced microscale surface irregularities. During the intermediate disintegration stage (Figure 11(a4–a6),(b4–b6),(c4–c6),(d4–d6)), the thickness of capsules in all media increased with an accelerated trend, with more pronounced swelling observed in pH = 4.5 and pH = 6.8 media. The images of the surface revealed the formation of distinct porous structures. Near the end of dissolution (Figure 11(a7–a9),(b7–b9),(c7–c9),(d7–d9)), the capsules exhibited distinct disintegration behaviors in different media. In the pH = 1.2 media, the thickness of capsules reached its maximum value, with large honeycomb-like pores appearing on the surface. These results can be attributed to the absence of potassium and sodium ions in the solution, where the concentration gradient of these ions, combined with the presence of HCl, promoted the outflow of potassium ions from the capsules, leading to the formation and inward expansion of honeycomb structures until complete dissolution. In the pH = 4.5 media, the thickness of the capsules decreased slightly at the end of dissolution, and a lamellar structure was observed on the surface. These results might be attributed to the presence of sodium ions in the solution, which inhibited the outflow of sodium ions from the capsules. The capsules could undergo swelling-driven disintegration, characterized by substantial swelling followed by delamination and dissolution in pH = 4.5 media. In the pH 6.8 media, the disintegration behaviors of the capsules were similar to those in pH 4.5 media, with more significant swelling, which may be attributed to the presence of both potassium and sodium ions in the media, which inhibited the outflow of these ions from the membrane. In the pH = 7.0 media, the capsules exhibited limited swelling and a lower thickness at the final dissolution stage, which may be due to the absence of HCl in the media, lacking its promoting effect on ion exudation, compared to the pH = 1.2 media.

**Figure 12:**
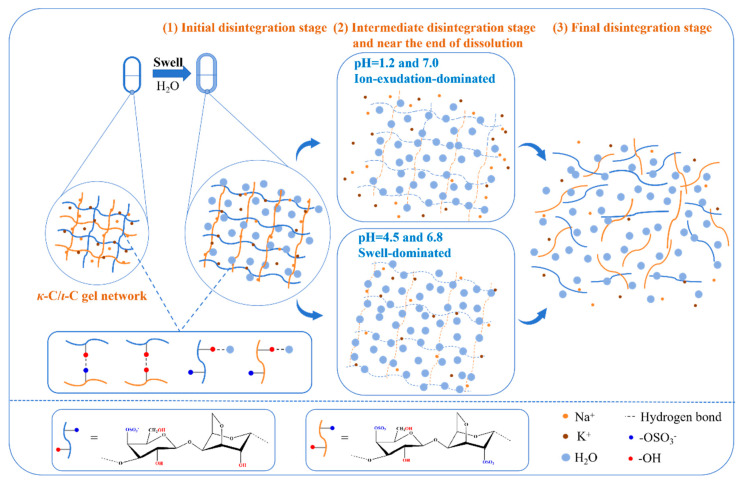
The hypothesis of the hydrogen bonding interaction evolution within the *κ*-C/*ι*-C gel network across varying physiological conditions.

In summary, the capsules exhibit two distinct pH-dependent disintegration processes: an ion exudation-dominated process under acidic (pH = 1.2) and neutral (pH = 7.0) conditions, characterized by rapid pore formation and structural collapse leading to accelerated disintegration, contrasted with a swelling-controlled mechanism under weakly acidic and neutral conditions (pH = 4.5 and 6.8) involving progressive hydration-induced thickness expansion and lamellar fragmentation that results in prolonged disintegration time. This work proposed a hypothesis that delineated the pH-dependent reorganization of hydrogen bonding networks in the polysaccharide gel network across varying physiological conditions (pH = 1.2, 4.5, 6.8, and 7.0), presented in Figure 12. At the initial disintegration stage, the interchain hydrogen bonding network formed between *κ*-C and *ι*-C polysaccharides could exhibit dynamic destabilization and structural reconfiguration, allowing for the preferential establishment of new hydration interactions with surrounding water molecules. Thus, *ι*-C could exhibit greater susceptibility to this transformation owing to its sulfate group content. The deprotonation of sulfate groups also induces the destabilization of hydrogen bonds between polysaccharide chains, as demonstrated in Section 3.8. Then, at the intermediate disintegration stage and near the end of dissolution in pH 1.2 and 7.0 media, the capsule continued to swell, accompanied by ion diffusion into the surrounding solution. This process led to the formation and inward expansion of honeycomb-like structures, ultimately resulting in complete dissolution. In contrast, in pH = 6.8 and 7.0 media during the same phase, the capsules exhibited a more pronounced swelling behavior, followed by swelling-driven disintegration, which progressively developed into delamination and ultimately led to dissolution. The final stage of disintegration involves the complete collapse of the gel network, accompanied by the dissolution and subsequent release of polysaccharide molecular chains into the surrounding media. This dynamic hydrogen bonding reorganization is postulated to underlie the observed pH-dependent disintegration profiles.

### 2.10. Properties of κ-C/ι-C Capsules

**Figure 13:**
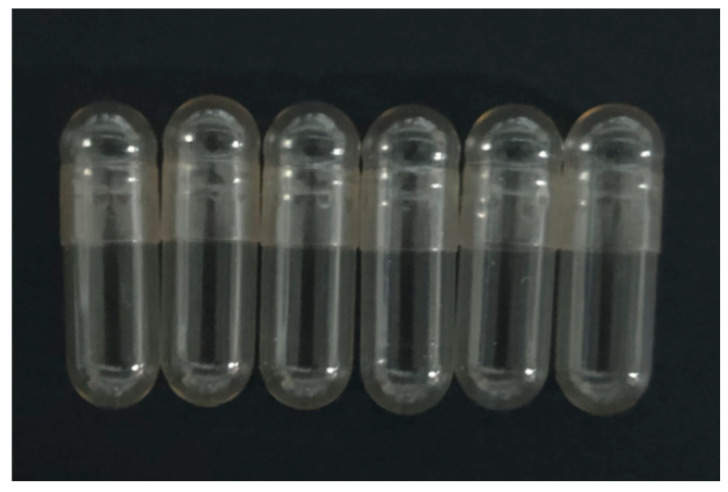
Photograph of *κ*-C/*ι*-C (5:5) HPMC-based hard capsules.

Through the above results, the selection of the NaCl + KCl mixed coagulant with a mass fraction of 0.16% and a KCl/NaCl ratio of 0.06:0.10, and a gelling agent with a *κ*-C/*ι*-C ratio of 5:5 enables the preparation of HPMC-based hard capsules with excellent mechanical properties and fast disintegration in four media (pH = 1.2, 4.5, 6.8, and 7.0). The prepared capsules are shown in Figure 13. The capsules are smooth and intact, with uniform color, high transparency, no plum blossom-shaped defects, no odor or taste, and no softening, deformation, or black spots. According to the methods of the Chinese Pharmacopoeia and the US Pharmacopeia [63], the purchased HPMC-based hard capsules, gelatin capsules, and the prepared *κ*-C/*ι*-C HPMC-based hard capsules in this work were tested for relevant physicochemical properties, as shown in Table 2. The properties of *κ*-C/*ι*-C HPMC-based hard capsules are excellent and meet the requirements of Pharmacopoeia standards. These capsules have potential for industrial production and may have applications in pharmaceuticals.

**Figure 14:**
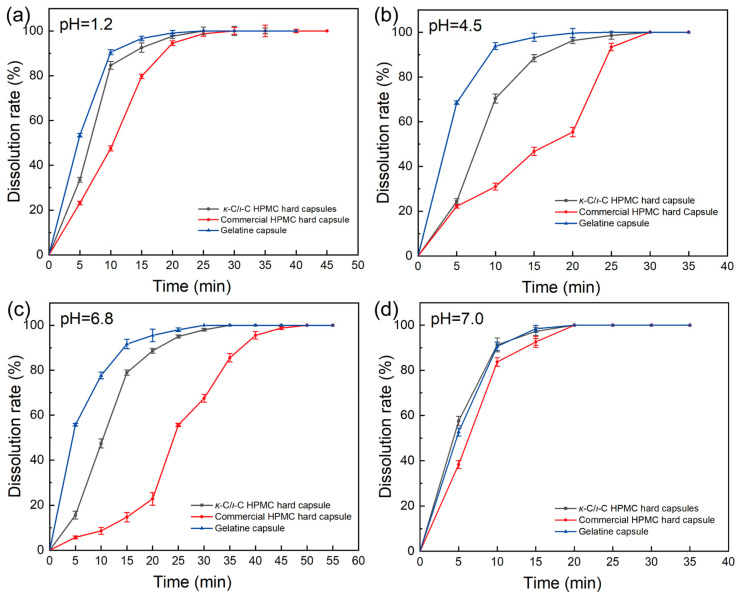
Dissolution curves of different types of hard capsules: (**a**) hydrochloric acid solution (pH = 1.2), (**b**) sodium acetate buffer (pH = 4.5), (**c**) phosphate buffer (pH = 6.8), and (**d**) deionized water (pH = 7.0).

The 5:5 *κ*-C/*ι*-C HPMC capsules were used for dissolution testing, with comparisons made against commercial HPMC capsules and gelatin capsules. To better reflect the degree of capsule dissolution and reduce interference from the dissolution of the drug itself in evaluating the dissolution performance of the capsules, ranitidine hydrochloride with good solubility was selected as the model drug for the dissolution study [64]. The experiment was carried out in four media at pH values of 1.2, 4.5, 6.8, and 7.0. The dissolution curve is shown in Figure 14. At pH 1.2, the dissolution of 5:5 *κ*-C/*ι*-C HPMC capsules in 15 min was 92.53 ± 2.1%, which was higher than that of other HPMC capsules (79.74 ± 0.98%) and close to that of gelatin capsules (96.64 ± 1.21%). At pH 4.5, the dissolution rate of the 5:5 *κ*-C/*ι*-C HPMC capsules was 88.41 ± 1.63%, which was much higher than that of other HPMC capsules (46.72 ± 1.84%) and close to that of gelatin capsules (97.74 ± 1.84%). At pH 6.8, the dissolution rate of the 5:5 *κ*-C/*ι*-C HPMC capsules was 78.95 ± 1.22%, which was significantly better than that of other HPMC capsules (14.74 ± 2.12%) and slightly lower than that of gelatin capsules (91.65 ± 2.11%). At pH 7.0, the dissolution rate of the 5:5 *κ*-C/*ι*-C HPMC capsules was 97.30 ± 1.82%, which was not significantly different from that of the gelatin capsules (98.45 ± 1.53%) and higher than that of other HPMC capsules (92.61 ± 2.51%). In addition, the dissolution of the 5:5 *κ*-C/*ι*-C capsules and gelatin capsules reached 70% within 15 min at pH levels of 1.2, 4.5, 6.8, and 7.0, which met the standard, indicating that the dissolution behavior of the two was comparable [15]. The dissolution performance confirmed that the 5:5 *κ*-C/*ι*-C HPMC capsules exhibited comparable drug release performance to gelatin capsules in various acid-base environments, indicating potential for drug delivery.

## 3. Materials and Methods

### 3.1. Materials

Food-grade hydroxypropyl methylcellulose (HPMC, ZW-HPMC-2910E4) was obtained from Huzhou Zhanwang Pharmaceutical Co., Ltd. (Huzhou, China), with a methoxy content of 29.0%, a hydroxypropoxy content of 8.2%, a viscosity of 4.1 mPa·s, and M_w_ of 2.65 × 10^4^ g/mol. *κ*-C (KA-80) and *ι*-C (IA-100G) were provided by Lvxin Foodstuff Co., Ltd. (Zhangzhou, China). *κ*-C (KA-80) has a sulfate content of 20.1%, a viscosity of 0.110 Pa·s, and an M_w_ of 6.69 × 10^5^ g/mol, while *ι*-C (IA-100G) has a sulfate content of 27.5%, a viscosity of 0.058 Pa·s, and an M_w_ of 4.37 × 10^5^ g/mol, with viscosity measurements taken at a 1.5% (*w*/*v*) concentration and 75 °C. Analytical-grade chemicals, including potassium chloride (KCl), sodium chloride (NaCl), and glycerol, were purchased from Xilong Scientific Co., Ltd. (Shantou, China). Commercial gelatin capsules were sourced from Fujian Shunchang Minyi Capsule Co., Ltd. (Nanping, China), and other plant-based capsules were obtained from Jiangsu LiFan Capsule Co., Ltd. (Zhenjiang, China).

### 3.2. Gel Solution Preparation

The optimal ratio of HPMC to glycerol, along with the total concentration of carrageenan (1%, *w*/*w*), was determined based on previous studies [65]. Initially, a 15% (*w*/*w*) HPMC solution was prepared by dissolving HPMC in deionized water at 80 °C under constant stirring to achieve a homogeneous mixture. Subsequently, 0.75% (*w*/*w*) glycerol was added to the solution. Various coagulants were tested, including commonly used types and their 1:1 mixtures. After complete dissolution, *κ*-C and *ι*-C were incorporated at a total concentration of 1% (*w*/*w*), with the following *κ*-C/*ι*-C weight ratios: 0:10, 3:7, 5:5, 7:3, 10:0. The resulting mixture was then stirred continuously at 80 °C for 4 h to ensure uniform polymer integration, followed by a temperature reduction to 60 °C and a 2 h standing period to facilitate gel formation.

### 3.3. Preparation of HPMC-Based Hard Capsules

The gel solution was cooled to 50 °C, and cylindrical stainless-steel molds (1# size, outer diameter: 7.5 mm; inner diameter: 7.1 mm) were immersed in the solution for 2 s. After removal, the molds were inverted several times to form a transparent, uniform gel layer. The coated molds were placed in a drying oven at 30 °C and 50% relative humidity for 4 h. After drying, the capsules were demolded, cut, and assembled. The thickness of the prepared HPMC-based capsules was controlled to 0.1 ± 0.005 mm.

### 3.4. Preparation of Membranes

The homogeneous gel solution was coated onto 10 × 10 cm^2^ glass plates, which were then placed in a drying oven at 30 °C and 50% relative humidity for 4 h. After drying, the membranes were peeled off, and their thickness was consistent with that of the prepared HPMC-based hard capsules.

### 3.5. Preparation of Different pH Media

To simulate the fasting gastric environment (pH = 1.2), a hydrochloric acid solution was prepared by diluting concentrated HCl with deionized water. The fed gastric environment (pH = 4.5) was modeled using a sodium acetate buffer prepared by diluting sodium acetate in deionized water. The intestinal environment (pH = 6.8) was simulated using a phosphate buffer prepared by dissolving potassium dihydrogen phosphate in deionized water and adjusting the pH with sodium hydroxide (NaOH). The colonic environment (pH = 7.0) was mimicked using deionized water without further adjustment.

### 3.6. Capsule Properties

#### 3.6.1. Mechanical Properties

The mechanical properties of the membranes were evaluated using an electronic tensile testing machine (CMT4204, Shanghai New Sans Enterprise Development Co., Ltd., Shanghai, China). Membranes were cut into strips measuring 10 mm × 80 mm. The initial grip separation was set to 50 mm, with a crosshead speed of 20 mm/min. Tensile strength (*σ*_M_) and elongation at break (*ε*_B_) were calculated from the average of three replicate tests using Equations (1) and (2):

(1) $$\sigma_{M}=\;F/A$$

(2) $${\;\varepsilon }_{B}=\;\left(L\;-\;L_{0}\right)/L_{0}\;\times \;100\%$$

where *F* (N) is the maximum force that the membrane can withstand at the moment of rupture, *A* (m^2^) is the original cross-sectional area of the membrane, *L* (mm) is the length of the membrane when it breaks, and *L*_0_ (mm) is the initial length of the membrane.

#### 3.6.2. UV-vis Measurements

The transmittance of the membranes was measured using a UV spectrophotometer (UV-8000, Shanghai Metash Instruments Co., Ltd., Shanghai, China). The membranes were cut into strips measuring 2 cm × 4 cm and placed in the sample holder. Measurements were taken at a wavelength of 600 nm. For each sample group, three replicates were conducted, and the average value was recorded.

#### 3.6.3. Water Content

The capsules, with approximately a weight of 3 g, were cut into 3 × 3 mm pieces and then evenly arranged in a flat weighing bottle. The initial mass was recorded, then the sample was dried at 105 °C for 5 h. After drying, the bottle lid was closed, and the sample was cooled in a desiccator for 30 min before reweighing. The drying and weighing process was repeated for 1 h intervals until the weight difference between two consecutive measurements was less than 5 mg. The water content (%) of the sample was calculated based on the weight loss using Equation (3):

(3) $$\mathrm{Water}\;\mathrm{content}=\frac{W_{0}\;-\;W_{1}}{W_{0}}\;\times \;100\%$$

where *W*_0_ is the initial weight of the sample (before drying), and *W*_1_ is the weight of the sample after drying.

#### 3.6.4. Disintegration Time

According to Chinese Pharmacopoeia (2020 edition), six capsules filled with talcum powder were placed in a rotating basket with a baffle. A total of 600 mL of artificial gastric fluid was poured into a 1000 mL beaker, which was positioned in an Intelligent Disintegration Tester (ZB-1E, Tianda Tianfa Technology Co., Ltd., Tianjin, China). The capsules were immersed in the fluid, and the disintegration time for all six capsules was recorded.

#### 3.6.5. Water Vapor Permeability (WVP)

The water vapor permeability of the membranes was determined using the cup method [66]. Circular disks with 7 cm diameters were placed over permeation cups containing 5 g of anhydrous calcium chloride (CaCl_2_) and sealed with a paraffin mixture. The cups were placed in a constant temperature and humidity chamber at 23 °C and 50% relative humidity. Weight gain was recorded every 12 h until the difference between two successive measurements was less than 5%. WVP was calculated using the final weight and exposure time. Each measurement was performed in triplicate, and the average value was reported. The equation for WVP calculation is shown in Equation (4):

(4) $$\mathrm{WVP}=\frac{\Delta m\cdot d}{A\cdot t\cdot \Delta p}$$

where Δ*m* (g) is the mass increase in the cup before, and after the test, *d* (m) is the thickness of membranes, *A* (m^2^) is the area of water vapor through the membrane, *t* (h) is the time taken for the membrane quality to change to a stable state, and Δ*P* (Pa) is the partial pressure difference across the membranes.

#### 3.6.6. Peroxide Value (PV)

Equal-mass aliquots of fresh soybean oil were transferred into glass test tubes and sealed hermetically with the test membranes. The tubes were then placed in a controlled temperature–humidity chamber maintained at 25 °C and 0% relative humidity for 12 days to induce oxidation. Peroxide values of the oxidized soybean oil were determined by titration with a 0.01016 mol/L standard sodium thiosulfate (Na_2_S_2_O_3_) solution. Each test was performed in triplicate, and the average value was recorded. The peroxide value was calculated using Equation (5):

(5) $$\mathrm{PV}=\frac{\left(V_{1}\;-\;V_{2}\right)\cdot c\cdot 0.1269}{m}$$

where *V*_1_ (mL) is the volume of the Na_2_S_2_O_3_ standard solution consumed by the membrane, *V*_2_ (mL) is the volume of the Na_2_S_2_O_3_ standard solution consumed by the blank, *c* (mol/L) is the concentration of the Na_2_S_2_O_3_ standard solution, *m* (g) is the mass of the sample, and 0.1269 (g) is the mass of iodine contained in 1 mL of the Na_2_S_2_O_3_ standard solution.

#### 3.6.7. Hygroscopicity Properties

After drying at 105 °C to a constant weight, the capsules were placed in hermetically sealed glass desiccators containing a saturated sodium chloride solution (75% relative humidity). Weight was recorded at 0, 0.5, 1, 6, 12, 24, 36, 48, 60, and 72 h. Each measurement was performed in triplicate, and the average value was reported. The hygroscopicity rate was calculated using Equation (6):

(6) $$\mathrm{Hygroscopicity}\;\mathrm{rate}=\frac{m\;-\;m_{0}}{m_{0}}\;\times \;100\%$$

where *m*_0_ (g) is the initial mass of the capsules after drying, and *m* (g) is the mass of the capsules at each sampling time point.

#### 3.6.8. Dissolution Rate

Ranitidine hydrochloride was selected as the model drug and then loaded into gelatin capsules, *κ*-C/*ι*-C HPMC-based hard capsules, and other HPMC capsules for dissolution testing according to the Chinese Pharmacopoeia (2020 edition). The dissolution media consisted of 900 mL artificial gastric juice (pH 1.2), acetic acid–sodium acetate buffer (pH 4.5), phosphate buffer (pH 6.8), and water. The temperature of the dissolution apparatus was set at 37 ± 0.5 °C, and the rotation speed was 50 ± 1 r/min. Samples were collected at 5 min intervals, filtered through a 0.22 μm membrane, and analyzed for absorbance at 314 nm using UV spectrophotometry (UV-8000, Shanghai Metash Instruments Co., Ltd., Shanghai, China). The test was terminated once absorbance readings stabilized. After each sampling, an equivalent volume of fresh dissolution medium was immediately replenished into the dissolution vessel. Drug dissolution was calculated based on the corresponding standard curve, which is presented below:

pH = 1.2 y = 0.0017x + 0.0177  R^2^ = 0.999

pH = 4.5 y = 0.0080x + 0.0134  R^2^ = 0.997

pH = 6.8 y = 0.0038x + 0.0058  R^2^ = 0.999

pH = 4.5 y = 0.0080x + 0.0134  R^2^ = 0.999

#### 3.6.9. Capsule Friability

The friability of the capsules was determined according to the testing method for HPMC-based hard capsules in the Chinese Pharmacopoeia. Equation (7) is as follows:

Friability (%) = (number of ruptured capsules/50) × 100%(7)

#### 3.6.10. Capsule Formation Rate

The capsule formation rate is defined as the ratio of the number of intact capsules successfully formed to the theoretical maximum number of capsules that could be formed during the preparation process. Equation (8) is as follows:

Capsule formation rate (%) = (number of intact capsules/numbers of theoretical capsules) × 100%(8)

### 3.7. Characterization

#### 3.7.1. Rheology

Rheological testing was conducted using an Anton Paar rotational rheometer (MCR-302, Anton Paar Shanghai Trading Co., Ltd., Shanghai, China). First, the temperature of the sample platform was raised to 50 °C. Then, 5–8 mL of the gel solution was aspirated with a pipette and evenly spread on the preheated sample platform. The distance between the flat plate and the sample platform was set to 1.0 mm, and the shear rate of the sensor was set in the range of 0.01–600.00 s^−1^. The flow curve of the gel solution was recorded and fitted using the Power-aw model [45,67,68]:

(9) $$\sigma =K\cdot {\gamma \;}^{n}$$

where *σ* (Pa) is shear stress, *K* (Pa·s^n^) is the consistency coefficient, *γ* (1/s) is the shear rate, and *n* is the flow behavior index.

#### 3.7.2. FTIR

The membrane samples were first subjected to freeze-drying. Immediately afterward, the samples were transferred to a desiccator containing anhydrous calcium chloride to minimize the impact of moisture as much as possible. FTIR spectroscopy analysis was performed using a spectrometer (NICOLET iS50, Thermo Fisher Scientific Co., Ltd., Shanghai, China) in the range of 4000–400 cm^−1^ with 32 scans.

#### 3.7.3. SEM

Freeze-dried membranes were prepared by casting the gel solution and subsequently freeze-dried in a lyophilizer. The freeze-dried samples were immersed in liquid nitrogen for 2 min and immediately fractured into fragments (~3 × 3 mm) using tweezers to expose the cross-sectional structure. Both surface and cross-section specimens were mounted on conductive adhesive-coated platforms, sputter-coated with gold, and observed using a scanning electron microscope (SU-5000, Hitachi High-Tech Corporation, Tokyo, Japan).

#### 3.7.4. TG-DTG

The membrane was dried in an oven at 105 °C until a constant weight was achieved, and then it was cut into small pieces. Approximately 5–10 mg of the sample was placed in an alumina crucible and analyzed using a thermogravimetric analyzer (TGA-50H, Shimadzu Corporation, Kyoto, Japan) to evaluate its thermal stability and decomposition behavior. The temperature was programmed to increase from 25 °C to 800 °C at a heating rate of 10 °C/min. The analysis was conducted under a high-purity argon atmosphere with a flow rate of 50 mL/min. Thermogravimetric (TG) curves were recorded following the test.

### 3.8. Statistical Analysis

The results were shown as the mean value ± standard deviation (S.D.). The results were subjected to analysis of variance (ANOVA) using the SPSS software (version 26) package (IBM, Armonk, NY, USA) with a significance level of *p* < 0.05.

## 4. Conclusions

This study developed HPMC-based hard capsules with excellent mechanical properties and fast disintegration across four pH media by employing a composite commercial gelling system consisting of *κ*-C and *ι*-C, with KCl and NaCl as coagulants. The prepared capsules, with a KCl/NaCl mass ratio of 0.06:0.10 at a total mass fraction of 0.16% and a *κ*-C/*ι*-C ratio of 5:5, exhibited optimal mechanical properties and transmittance. FTIR, SEM, and TGA revealed that capsules at a 5:5 ratio (*κ*-C/*ι*-C) exhibited optimized synergistic effects and a dense gel network structure, providing strong mechanical support for the capsules and enhancing their disintegration performance. WVP, PV, and hygroscopicity properties revealed that this ratio was optimal for both storage stability and drug stability. Finally, SEM was employed to track the swelling and disintegration behaviors in different pH media, thereby inferring the possible disintegration mechanism.

In summary, the *κ*-C/*ι*-C composite (5:5 ratio) can serve as an excellent gelling agent, enabling fast disintegration across four pH media. This study proposes an innovative approach to addressing the pharmaceutical challenge of achieving rapid drug release, demonstrating significant potential and thereby broadening the application of plant-based capsules in the field of medicine.

## Figures and Tables

**Table 1:** The fitting parameters of the Power-law model based on the shear stress–shear rate curves.

*κ*-C/*ι*-C	*K*/Pa·s^n^	*n*	*R* ^2^
0:10	0.4218 ± 0.0348	0.7615 ± 0.0137	0.9905
3:7	0.4989 ± 0.0062	0.7884 ± 0.0021	0.9998
5:5	1.7440 ± 0.0600	0.6576 ± 0.0057	0.9978
7:3	1.9108 ± 0.0833	0.6547 ± 0.0073	0.9965
10:0	5.0277 ± 0.2037	0.5473 ±0.0068	0.9954

**Table 2:** Comparison of physicochemical properties of *κ*-C/*ι*-C HPMC-based hard capsules, HPMC hard capsules, and gelatin capsules.

Samples	This Work	Purchased Gelatine Capsules	Purchased HPMC-Based Hard Capsules	The Pharmacopoeia Requirements for HPMC-Based Hard Capsules
Hardness/N	30.44 ± 1.01	39.54 ± 3.12	29.37 ± 0.32	-
Resilient/Pa	0.831 ± 0.017	0.815 ± 0.020	0.816 ± 0.024	-
Disintegration time (pH = 1.2)/min	<7	<6	<15	<15
Disintegration time (pH = 4.5)/min	<9	<9	<17	<15
Disintegration time (pH = 6.8)/min	<11	<7	<16	<15
Disintegration time (pH = 7.0)/min	<8	<8	<12	<15
Elasticity	0/10	0/10	0/10	≤1/10
Brittleness	0/50	0/50	0/50	≤2/50
Loss on drying/%	5.84 ± 0.30	13~17	<8	<8
Scorching residue/%	1.17	<1.50	<3	<3

## Data Availability

The datasets used and/or analyzed during the current study are available from the corresponding author on reasonable request.

## Supplementary Materials

The following supporting information can be downloaded at: https://www.mdpi.com/article/10.3390/md23070284/s1, Figure S1: Porosity of *κ*-C/*ι*-C capsules; Figure S2: Electron microscope images of the initial section and surface of the membrane.

## Abbreviations

The following abbreviations are used in this manuscript:

*κ*-C	Kappa-Carrageenan
*ι*-C	Iota-Carrageenan
HPMC	Hydroxypropyl methylcellulose
WPV	Water vapor permeability
PV	Peroxide value
SEM	Scanning electron microscope
TG-DTG	Thermogravimetric analysis–derivative thermogravimetry
