# Supplementary material for: κ/ι-Carrageenan Blends in Plant Capsules: Achieving Harmony Between Mechanical and Disintegration Properties
Source: Mar Drugs. 2025 Jul 9;23(7):284. doi: 10.3390/md23070284 (PMC12298366; doi:10.3390/md23070284)
Supplement: Supplementary file 1 [file marinedrugs-23-00284-s001.zip › marinedrugs-3737931-supplementary.pdf]

Supplementary file

κ/*ι*-Carrageenan Blends in Plant Capsules: Achieving Mechanical and Disintegration Properties Harmony

Zhenyu Liu[a,b#], Chuqi He[a,b#], Zhibin Yang[a], Qing Zhao[a], Yuting Dong[a,b], Jing Ye[a,b], Bingde Zheng[a,b], Ranjith Kankala[a], Xueqin Zhang[a,b##], And Meitian Xiaoa,b[##]

a College of Chemical Engineering, Huaqiao University, Xiamen 361021, Fujian Province, China;

b Xiamen Engineering and Technological Research Center for Comprehensive Utilization of Marine Biological Resources, Xiamen 361021, Fujian, China;

Correspondence: Xueqin Zhang xqzhang2009@hqu.edu.en; Tel.:+86 592 6162300 ; Meitian Xiao mtxiao@hqu.edu.en; Tel.:+ 86 592 6162300 .

# Contributed equally

1. Porosity of *κ*-C/*ι*-C Capsule Membrane

The smaller the porosity, the higher the compactness. The porosity of capsules was calculated from the SEM images (Figure 9). As shown in Figure S1, the capsules exhibited the smallest porosity of 31.98 ± 7.42 % when the *κ*-C/*ι*-C ratio was 0:10, followed by the 5:5 ratio with a porosity of 51.76 ± 2.22 %.

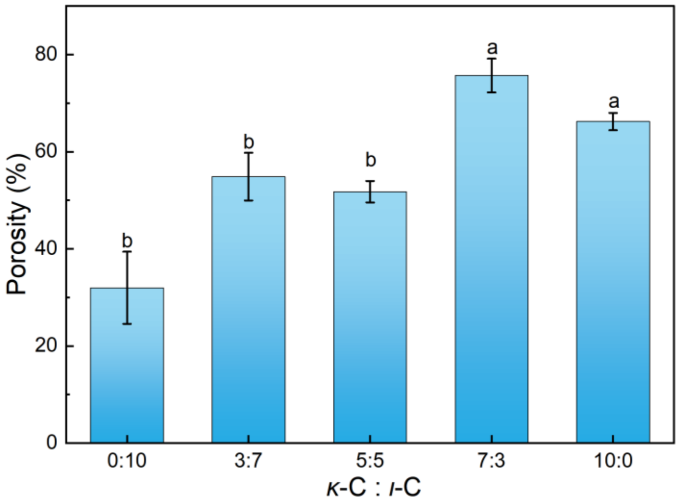

Figure S1 Porosity of *κ*-C/*ι*-C capsules

2. SEM images of the initial section and surface of the membrane

As shown in Figure S2, it is a microstructural image depicting the initial surface and section of the membrane, with the section thickness of the capsule (t = 0 min) measured to be 96.62 mm. The surface is smooth without cracks.

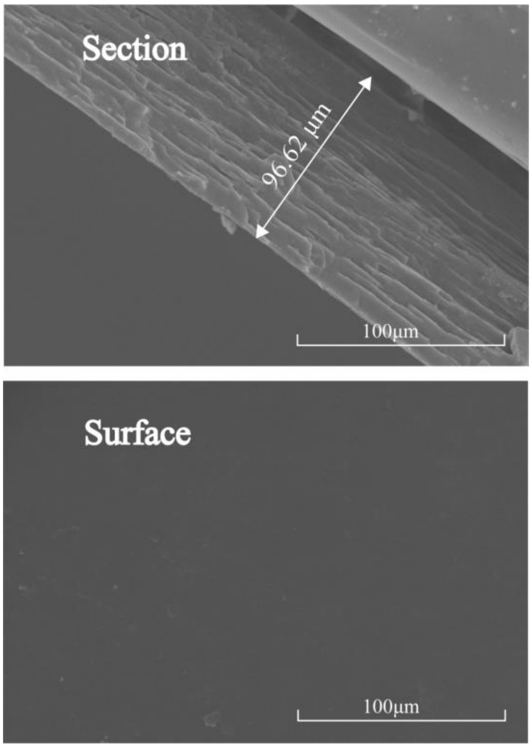

Figure S2 Electron microscope images of the initial section and surface of the membrane
